# Load Eccentricity of Compressed Composite Z-Columns in Non-Linear State

**DOI:** 10.3390/ma15217631

**Published:** 2022-10-30

**Authors:** Pawel Wysmulski

**Affiliations:** Department of Machine Design and Mechatronics, Faculty of Mechanical Engineering, Lublin University of Technology, 36 Nadbystrzycka Street, 20-618 Lublin, Poland; p.wysmulski@pollub.pl

**Keywords:** thin-walled structures, laminates, eccentricity of load, postcritical state, finite element method

## Abstract

The study investigated short, thin-walled Z-shaped carbon–epoxy laminate columns. Z-columns were compressed while considering the eccentric force realized from the center of gravity of the column section. The study involved performing a nonlinear analysis of the structures with implemented geometric imperfections reflecting the first buckling modes. The nonlinear analysis was performed by using the Tsai–Wu criterion to determine the effort of the composite material. The computations were run until the critical parameter was reached in the Tsai–Wu criterion, allowing for a description of the failure initiation mechanism in the composite material. The first signs of damage to the composite material were determined by using the acoustic emission method. Based on the results, postcritical equilibrium paths of the numerical models were determined. The equilibrium paths were then compared with the experimental characteristics of real structures. The numerical results and experimental findings show a satisfactory agreement. The results confirmed that the numerical models were adequate for estimating the performance of composite structures in the postcritical range, depending on the amplitude of compressive load eccentricity. The research topic undertaken is important because the thin-walled structure design relates to actual loads which, in most cases, differ from the idealized theoretical load conditions.

## 1. Introduction

Modern thin-walled contractions are mainly made of advanced composite materials characterized by low specific weight and high mechanical parameters. It mainly concerns thin-walled stiffeners [1,2,3,4,5,6,7], which are usually designed as open and closed profiles with complicated cross-section shapes. The role of such structures is to carry axial and bending loads, meaning that they are subject to buckling, particularly in compression. However, although the loss of stability of these members may result in a localized weakening of the load-bearing structure, it does not affect the immediate risks to the safe use of such a construction [8,9,10,11]. Available research has shown that thin-walled composite members can still carry loads in the buckling state, provided that the buckling is elastic and the equilibrium path after buckling is stable [12,13,14,15,16,17,18,19,20,21,22]. Therefore, in addition to investigating member buckling, it is also necessary to investigate the buckling behavior of the entire structure and its buckling capacity. Loss of stability in thin-walled stiffeners usually takes the form of local buckling of the walls and web of the thin-walled column and is manifested by the occurrence of a certain number of half-waves along the column axis [23,24,25,26,27,28]. Thin-walled composite structures of this type are distinguished by the fact that they are able to continue to carry the load after the critical load has been exceeded, provided that their postcritical characteristics are stable. From this fact, the possibility of describing the workings of the structure in a post-critical state is of great importance, both for cognitive [29,30,31,32,33,34,35,36] and application aspects.

An additional noteworthy aspect of the design of thin-walled structures concerns the real loads, which, in most instances, deviate from the idealized, theoretical loading conditions. A very significant problem in this field is the possibility of load eccentricity resulting, for example, from inaccurate installation or unstable boundary conditions. This is important because the response of a structure operating under eccentric loading conditions differs significantly from the idealized response to axial loading. Eccentricity of load application can result in premature loss of stability, with buckling in the service load range [37,38]. Under the influence of load eccentricity, changes may occur in the way the column walls are loaded, as the eccentricity of force application generates an additional flexural load condition, which may contribute to accelerated buckling. If the structure enters buckling too early, the strength performance of the structure is reduced, and this can lead to a more rapid failure of the loaded structures. Most design solutions do not take this phenomenon into account at the design stage, making it more dangerous to the actual performance of the structure.

This study aimed to determine qualitative and quantitative effects of compressive load eccentricity on the behavior of thin-walled composite columns in the postcritical state up to the first signs of failure of the composite structure. Different load eccentricity values were tested by numerical and experimental methods. The manuscript presents a novel approach for determining the load initiating failure to first composite layer. This consisted of using AEM (acoustic emission method) in the experiment, with which the first indication of damage in the composite was recorded. The experimental study was compared with an FEM analysis, with which the damage-initiating force of the first layer was determined by using the well-known Tsai–Wu criterion.

The finite element method (FEM) was chosen for the numerical analysis. FEM is currently one of the most widely used computational methods for the analysis of solid structures with a large number of degrees of freedom. Commercially available commercial FEM calculation packages allow for simple linear analyses, as well as complex calculations involving geometrically and physically non-linear issues [39,40,41,42,43,44,45,46]. It is also worth mentioning that this study involved using the popular finite element method, which is widely used in many fields [47,48,49,50,51,52,53,54,55].

## 2. The Test Object

**Figure 1:**
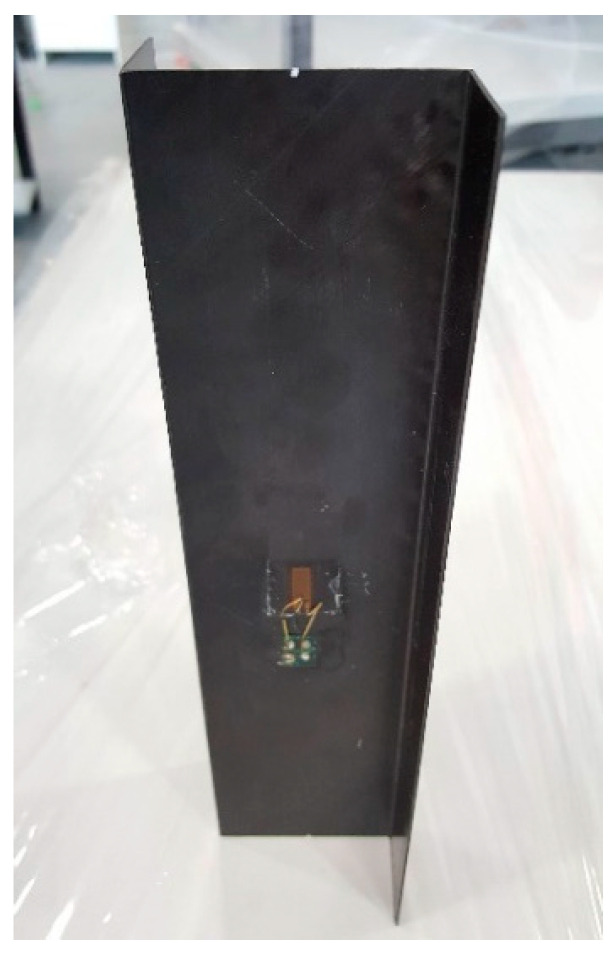
Real object of the study made of CFRP.

Short, thin-walled Z-shaped carbon–epoxy laminate columns were axially and eccentricity loaded in compression. Columns were standard thin-walled constructions with perpendicular walls. Every column consisted of three flat plate elements that were joined together [56,57,58,59,60,61]. The column material was an eight-layered carbon–epoxy laminate manufactured by autoclave (Figure 1).

Thin-walled columns were made from a unidirectional prepreg strip of the HexPly system carbon–epoxy composite with the designation M12/35%/UD134/AS7. The matrix of the composite was epoxy resin (density: ρ_(m)_ = 1.24 g/cm^3^; T_g(m)_ = 128 °C; R_m(m)_ = 64 MPa; ν_(m)_ = 0.4; E_(m)_ = 5.1 GPa), while the reinforcement was AS7J12K carbon fibers (density: ρ_(f)_ = 2.5 g/cm^3^; R_m(f)_ = 4830 MPa; ν_(f)_ = 0.269; E_(f)_ = 241 GPa). The nominal volume proportion of reinforcing fibers in the composite was approximately 60%.

The manufacturing process involved the preparation of a hermetic vacuum package in a special air-conditioned “clean room” on a prepared mold, enabling the dimensions and shape of the profiles to be reproduced. The fabricated vacuum package was connected to a vacuum pump, providing a vacuum of approximately 0.08 MPa, and then subjected to a polymerization process in an autoclave. The curing process in the autoclave is achieved by a rapid temperature rise under controlled pressure, isothermal annealing for the time required for the process to take place, and then cooling down. The process parameters (vacuum in the package, overpressure, temperature, and process time) are selected individually depending on the composite being produced. For the carbon–epoxy composite, an overpressure value of 0.4 MPa in the autoclave and a heating temperature of 135 °C for about 2 h were assumed.

**Figure 2:**
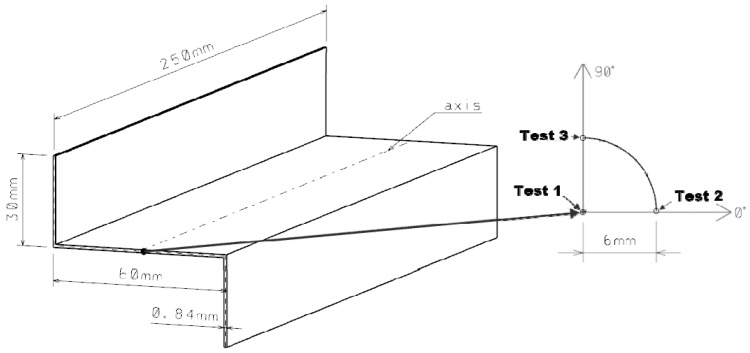
Geometrical Z-shaped column model with the schematic of the eccentricity load.

The composite Z-shaped columns had four layups, and they are shown in Table 1. The columns consisted of eight layers symmetrically arranged with respect to the central plane. The test object had the overall dimensions of the column cross-section: a web width of 60 mm, a wall width of 30 mm, and a length of 250 mm (Figure 2).

A schematic of a cross-section of a Z-shaped construction under the eccentric compressive load was presented in Figure 2, demonstrating that eccentricity is caused by a shift in the point of application of the compressive force with respect to the longitudinal axis of the column. The point was moved from center of gravity of the column (Test 1) to the 0° axis by a value of 6 mm (Test 2), and then the column was rotated by 90° (Test 3), with its midpoint located in the center of gravity of the column, as shown in Figure 2. The mechanical properties (Young’s modulus parallel to fibers, E_1_; Young’s modulus normal to fibers, E_2_; the Poisson’s ratio in the layer plane, ν_12_; and Kirchhoff’s modulus, G_12_) and limit properties (Tensile Strength parallel to fibers, F_T1_; Tensile Strength normal to fibers, F_T2_; Shear Strength, F_S_; Compressive Strength parallel to fibers, F_C1_; and Compressive Strength normal to fibers, F_C2_) of a single composite ply shown in Table 2.

## 3. Methodology

The scope of the research carried out included the analysis of the postcritical state up to the load value corresponding to the failure initiation of the first laminate layer, considering the eccentricity of the compression load application. A detailed analysis of the critical state of this type of structure is presented in other works by the author [62]. Investigations were carried out experimentally, and a numerical analysis was performed [63]. The finite element method (FEM) was chosen for the numerical analysis. In this paper, the ABAQUS/CAE 2020 system, using the finite element method, was used for the numerical computations.

Load eccentricity tests on composite Z-columns were carried out up to a condition corresponding to the moment of initiation of composite damage. In order to investigate this, the time course of the force was recorded during the experimental tests and acoustic effects were measured by using the acoustic emission method (AEM), which can indicate the first signs of damage to the composite material. The result of the experimental research conducted was the identification of the form of deformation of composite columns and the determination of the value of the force initiating failure of the specimen for a given value of load eccentricity. In parallel, a non-linear numerical analysis was carried out to identify the failure initiation of compressed Z-columns, using the tensor failure criterion of Tsai–Wu composites. The load corresponding to the compression of the Tsai–Wu criterion was the numerical value of the force initiating the failure of the first laminate layer.

## 4. Experimental Test

**Figure 3:**
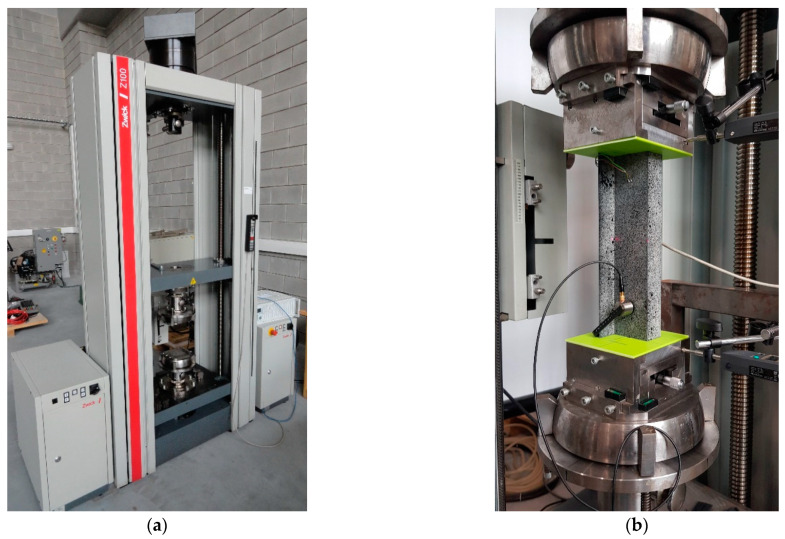
Experimental setup: (**a**) static testing machine and (**b**) test stand.

Experimental tests were carried out by using the static testing machine at 21 °C, with a constant displacement speed of the upper crosshead (Figure 3a). Figure 3b shows the test stand where experimental compression testing of thin-walled composite Z-columns was carried out.

In the experimental study, wall displacements of the Z-column were measured in the direction perpendicular to the wall plane, using the laser sensor. The results of the measurements carried out made it possible to develop postcritical equilibrium paths that allow the behavior of the columns to be described in terms of the compression load realized. Special clamping heads mounted on the pins of the testing machine were used to ensure articulated support conditions for the column’s end sections, incorporating spherical hinges. In addition, shims of deformable polymer material were used to eliminate imperfections in the boundary conditions and to ensure uniform loading of the individual edges of the column end sections. Specially designed sliding tables were mounted on the clamping heads, which, with the use of a micrometric screw, allowed for the precise introduction of compression load eccentricity values by appropriately positioning the profile with relation to axis of the testing machine. The position of load eccentric was determined by moving the Z-column profile in the 0° axis by 6 mm relative to the axis of the testing machine and then rotating it by 90°.

## 5. Numerical Model

Numerical investigations using the FEM for non-linear analysis of the postcritical state were carried out up to the failure initiation value of the CFRP structure. The numerical value of the load initiating the failure of the first composite layer was determined based on the iterative failure criterion of Tsai–Wu [64]:

(1) $$F_{11}\sigma_{1}^{2}+2F_{12}\sigma_{1}\sigma_{2}+F_{22}\sigma_{2}^{2}+F_{66}\tau_{12}^{2}+F_{1}\sigma_{1}+F_{2}\sigma_{2}=1$$

where the components of the strength tensors with the exception of *F*_12_ could be determined by simple strength tests. The values of these components could be represented as follows:

(2) $$F_{1}=\frac{1}{X_{t}}-\frac{1}{X_{c}}$$

(3) $$F_{2}=\frac{1}{Y_{t}}-\frac{1}{Y_{c}}$$

(4) $$F_{11}=\frac{1}{X_{t}X_{c}}$$

(5) $$F_{22}=\frac{1}{Y_{t}Y_{c}}$$

(6) $$F_{66}=\frac{1}{S^{2}}$$

where *X_t_* is the tensile strength of the composite in the fiber direction, *X_c_* is the compression strength of the composite in the fiber direction, *Y_t_* is the tensile strength of the composite in the direction crossed to the fibers, *Y_c_* is the compressive strength of the composite in the direction crossed to fibers, and *S* is the shear strength of the composite in the plane of the layer (assumed to be *S = S’* the same value for shear in both directions).

**Figure 4:**
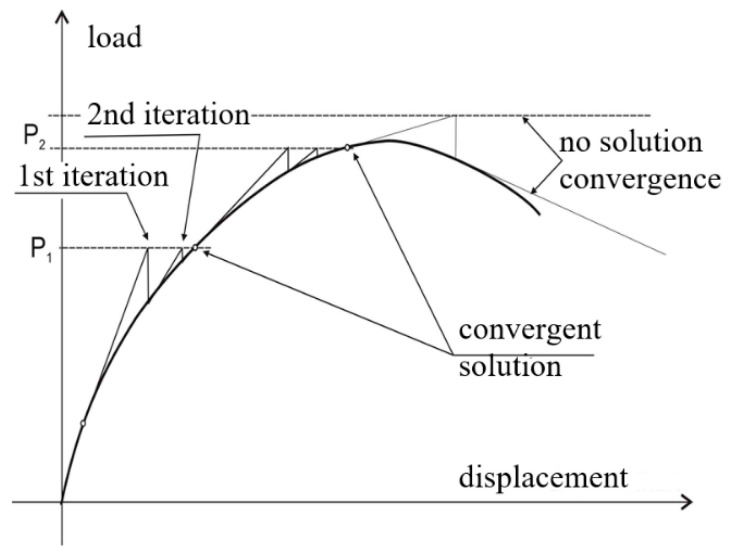
Newton–Raphson method.

The non-linear computation was carried out on a Z-column model with an implemented initial value of geometric imperfections corresponding to the obtained first buckling form of column, whose amplitude was *w_0C_* = 0.05 mm. The initial imperfection value used was determined by numerical validation of the postcritical equilibrium paths of the tested columns with the results of experimental tests. The non-linear stability solution was solved by using the incremental–iteration Newton–Raphson method (Figure 4).

**Figure 5:**
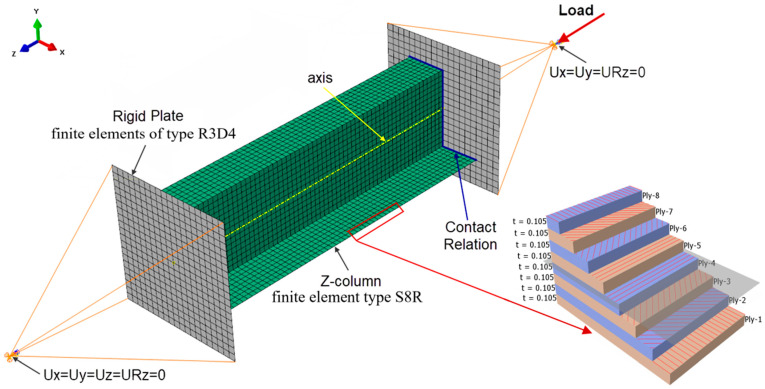
FEM model of the Z-column with boundary conditions and composite structure after the thickness of the shell element.

The discretization of the column was carried out by using SHELL-type layered shell elements, having three translational and three rotational degrees of freedom at each node of the element. In the developed numerical model of the column, a finite-element-type designated S8R was used, representing an 8-node element with a second-order shape function and reduced integration. In the case of the plates modeling the support planes of the column end sections, finite elements of type R3D4 were assumed, being rigid 4-node elements of type RIGID (Figure 5). The FEM numerical analysis did not consider large displacements. The numerical models prepared were characterized by a finite-element mesh structure with an individual element size of 4 mm × 4 mm. This allowed for a uniform partitioning of the individual profile walls by generating a constant density mesh. This approach enabled accurate observation of the deformation and stress states during the working of the compressed thin-walled Z-columns. The finite element size of the discrete model mesh was chosen after preliminary FEM analyses [65,66]. In addition, it should be added that there is the X-FEM (mesh-independent) method [67,68,69,70] in which the size of the finite element is not affected by the computational results. According to References [71,72,73,74,75], the FEM mesh size is of negligible significance in the X-FEM method.

The type of finite element used allowed for the definition of the layered composite structure along the vector normal to the surface of the element. The laminate structure was modeled by using the layup/ply modeling technique, which allows for the configuration of the composite layer structure to be modeled along the thickness of the finite element by defining the following parameters: layer thickness, type of material, and direction of fiber arrangement in the layer. The value of the thickness of a single laminate layer is t = 0.105 mm (total column wall thickness g = 0.84 mm), and this was determined from geometric measurements of the real composite structure. The numerical models developed defined an orthotropic material model in the plane stress state, based on the experimentally determined mechanical properties of the composite material, as shown in Table 2.

The boundary conditions of the FEM model reproduced the real support of the Z-column at both ends on the testing machine. The reference points used were those corresponding to the location of the centers of gravity of ball joints of the attachment heads, where simple support boundary conditions were defined. The two translational degrees of freedom (Ux = Uy = 0—Figure 5) and rotation about the column axis (URz = 0—Figure 5) were limited at the point reproducing the upper ball joint, while the three translational degrees of freedom (Ux = Uy = Uz = 0—Figure 5) and rotation (URz = 0—Figure 5) were limited at the reference point for the lower ball joint. Reference points were connected to rigid plates that support the ends of the columns by coupling all the degrees of freedom of the points and plates. The column was subjected to a compressive force concentrated at the upper reference point. The value of eccentricity parameter given was used by moving column from axial position (Test 1) toward the 0° axis by 6 mm in relation to the axis of the machine (Test 2) and then rotating it by 90° (Test 3). The contact relationship between the ends of the columns and the plates was defined.

## 6. Results

**Figure 6:**
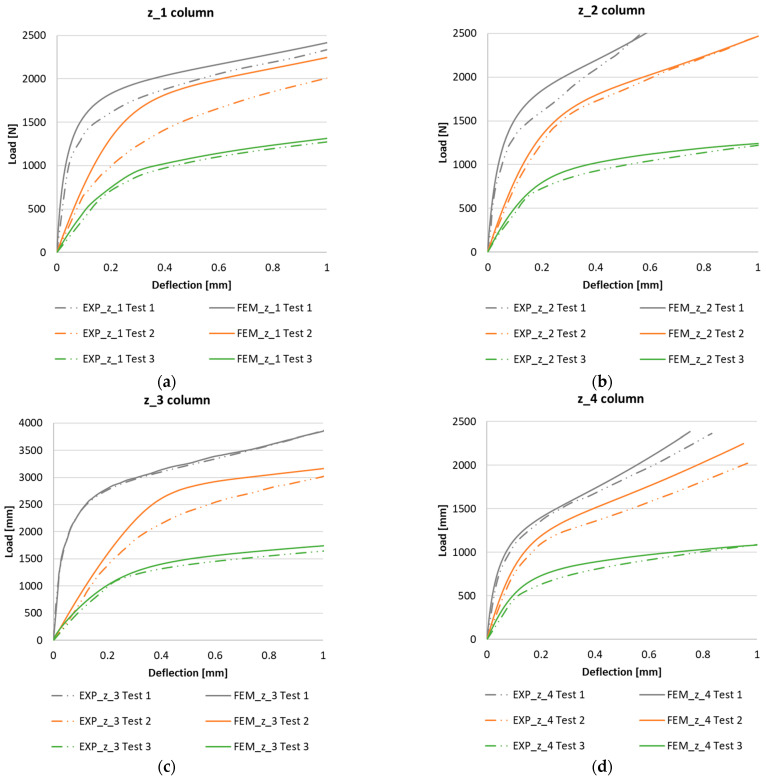
Postcritical EXP-FEM equilibrium paths in the realization of compressive load eccentricity: (**a**) z_1 column, (**b**) z_2 column, (**c**) z_3 column, and (**d**) z_4 column.

The analysis of the postcritical state was based on a comparison of the experimental and numerical postcritical equilibrium paths of the Z-column. Experimental compressive load: Increased deflection equilibrium paths were made on the basis of the recorded force and laser pointing perpendicularly to the half-wave formed on the wall of the Z-column, which was characterized by the largest deflections. Figure 6 shows a comparison between the experimental tests and the results of the numerical analysis of columns z_1÷z_4 for the case of axial compression (Test 1) and the cases of load eccentricity values realized in Test 2 and Test 3.

The characteristics obtained show a high agreement between the experimental results and the FEM numerical analysis. In both cases, the obtained characteristics have a similar stability character of work, and this confirms the ability of the structure to continue to carry the load in the postcritical range. The determined postcritical equilibrium paths of the compressed columns with Z-shape sections for all the configurations tested show a tendency for the stiffness of the structure to decrease due to the introduction of the load eccentricity, as shown in Figure 6. In the case of the real structure, the postcritical equilibrium paths showed slightly lower stiffness than the numerically determined curves.

By subjecting the change in stiffness of the structure caused by the introduction of compressive load eccentricity to numerical analysis, it could be concluded that the level of reduction in the stiffness of the structure in the postcritical range varies depending on the position of implementation of the load eccentricity. The introduction of the load eccentricity of Test 2 reduced the rigidity of the structure (depending on the layout of the composite) in the range of 12÷22%, while in Test 3, it caused a reduction in stiffness that was higher and was in the range of 46÷64%. Table 3 shows the changes in the stiffness of the Z-column under eccentric loading for all the composite-layer-configuration variants considered. From the results, it could be seen that the eccentricity of the compressive load has the greatest effect on the performance of the real structure in the postcritical state for configuration z_2 a, while it has the least effect for configurations z_1.

The compression tests on columns with Z-shape cross-sections were carried out until the first signs that could indicate the initiation of failure in the composite material were recorded by using the acoustic emission method. The scope of the experimental investigations included conducting 2 tests for all laminate configurations analyzed, covering Test 1 (axial compression) and Test 3. The measurements carried out on the real specimens allowed verification of the results of the numerical analyses. In the AEM tests, the characteristics of the signal amplitude compared with the time and the force were used to assess the moment of initiation of damage to the composite material.

The objective of the numerical analysis was to determine the compressive load at which the initiation of failure of the first layer of the laminate structure occurs. The failure of the composite material was assessed on the basis of the Tsai–Wu composite failure criterion (tensor criterion) implemented in the ABAQUS program. Non-linear structural computations were performed on models with the first form of buckling initiated by using the incremental–iterative Newton–Raphson method. Numerical computations were performed until the failure criterion of reaching critical parameter 1 (on a scale of 0÷1) was reached. The regions of the structure in which the critical value of the failure parameter has been reached define critical zones for which there is a high probability of failure of the composite layer. The presented FEM results show the state of reaching the critical parameter in the first composite layer and the corresponding value of the force initiating the failure of the composite material.

**Figure 7:**
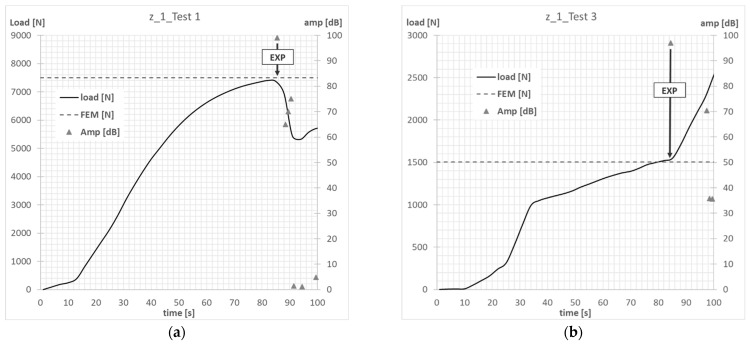
Column z_1—failure initiation force: (**a**) axial compression—Test 1; (**b**) eccentricity—Test 3.

**Figure 8:**
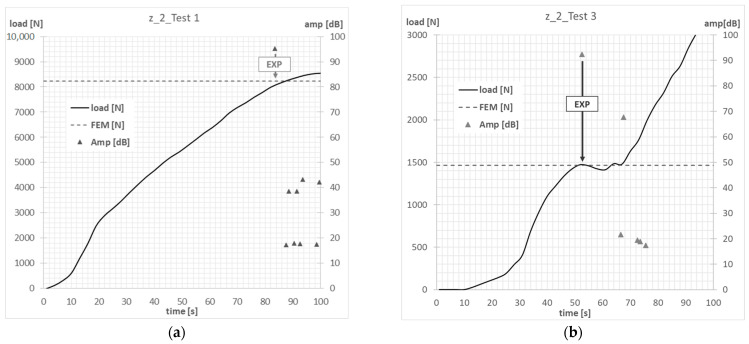
Column z_2—failure initiation force: (**a**) axial compression—Test 1; (**b**) eccentricity—Test 3.

**Figure 9:**
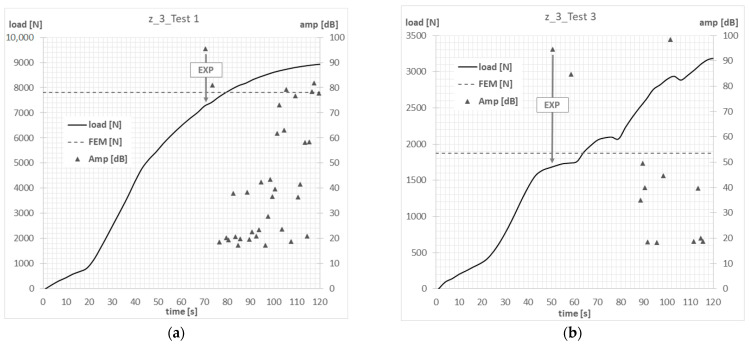
Column z_3—failure initiation force: (**a**) axial compression—Test 1; (**b**) eccentricity—Test 3.

**Figure 10:**
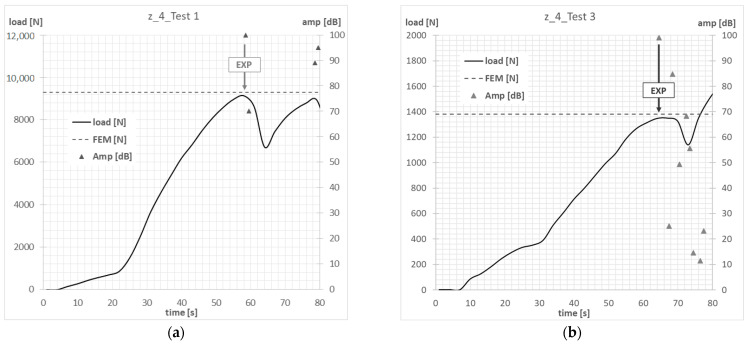
Column z_4—failure initiation force: (**a**) axial compression—Test 1; (**b**) eccentricity—Test 3.

The compressive-force waveform and the amplitude of the acoustic emission signal over time, determined during the experimental tests, allowed us to determine the value of load initiating failure of the first layer of composite of actual structure. The experimental value of the force initiating the failure of the composite material was determined on the basis of the first clear decrease in the waveform of the force in time, which was simultaneously accompanied by a clear increase in the amplitude of the EA signal. In the conducted tests, the experimental value of the force initiating failure was, in most cases, the maximum of the local extremum of the force-time characteristic. Figure 7, Figure 8, Figure 9 and Figure 10 show the methodology for the graphical determination of the experimental value of the force initiating failure of composite columns with Z-shape sections.

**Figure 11:**
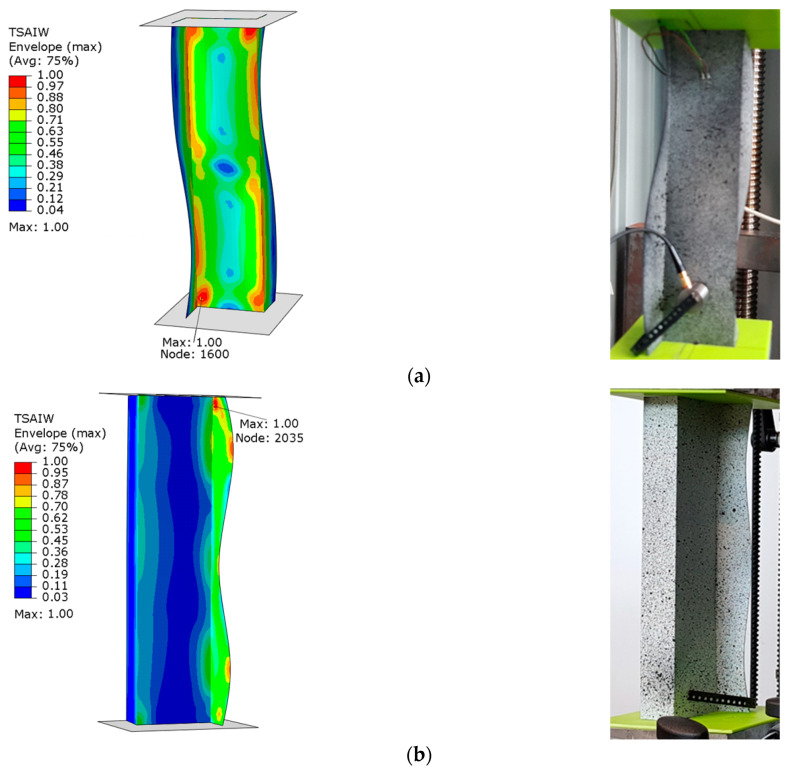
Column z_1—maps of the Tsai–Wu critical parameter and forms of post-critical deformation: (**a**) Test 1 and (**b**) Test 3.

**Figure 12:**
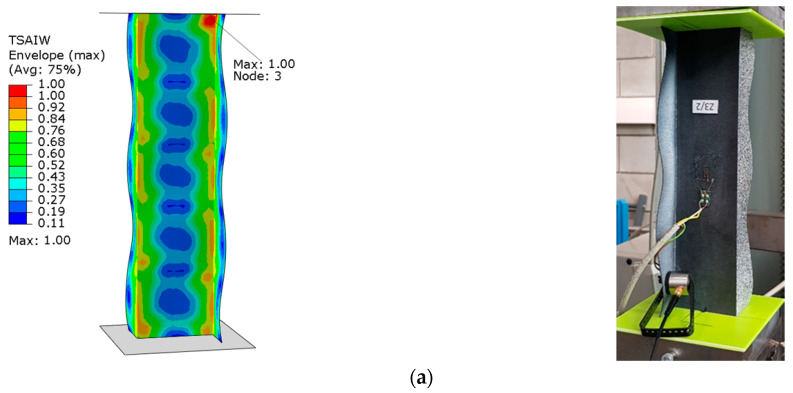

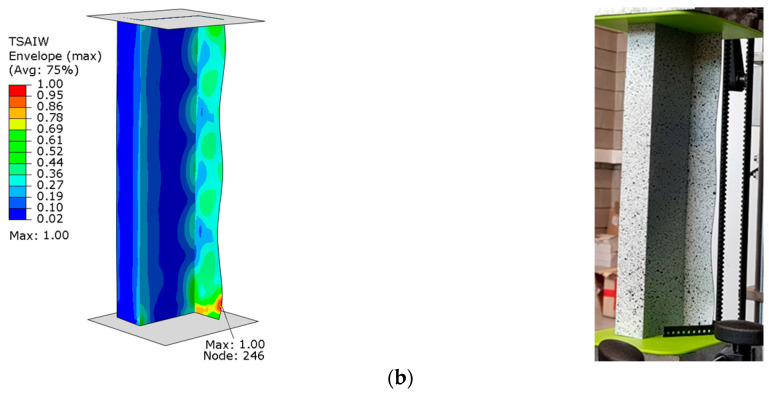
Column z_2—maps of the Tsai–Wu critical parameter and forms of post-critical deformation: (**a**) Test 1 and (**b**) Test 3.

**Figure 13:**
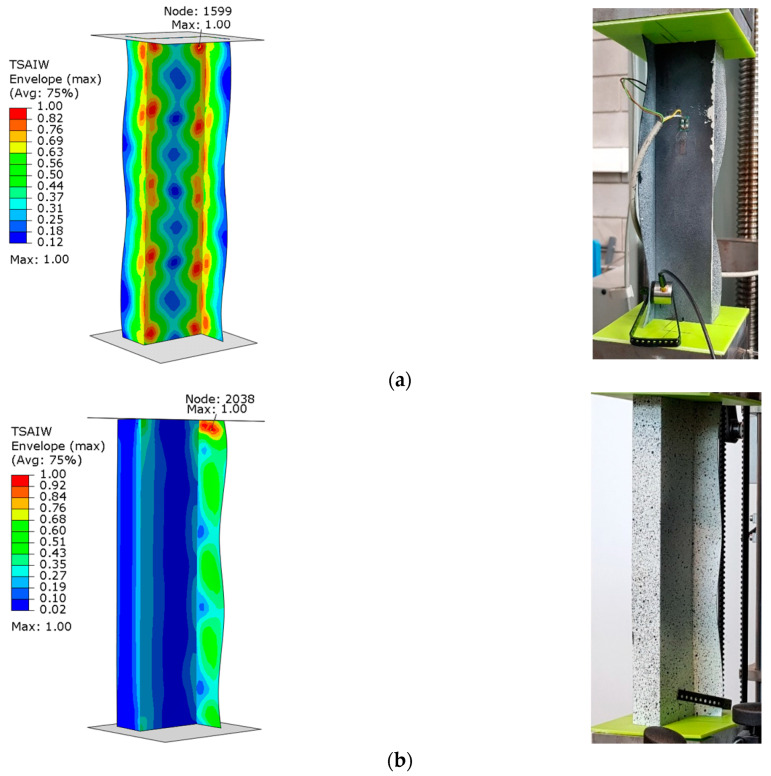
Column z_3—maps of the Tsai–Wu critical parameter and forms of post-critical deformation: (**a**) Test 1 and (**b**) Test 3.

**Figure 14:**
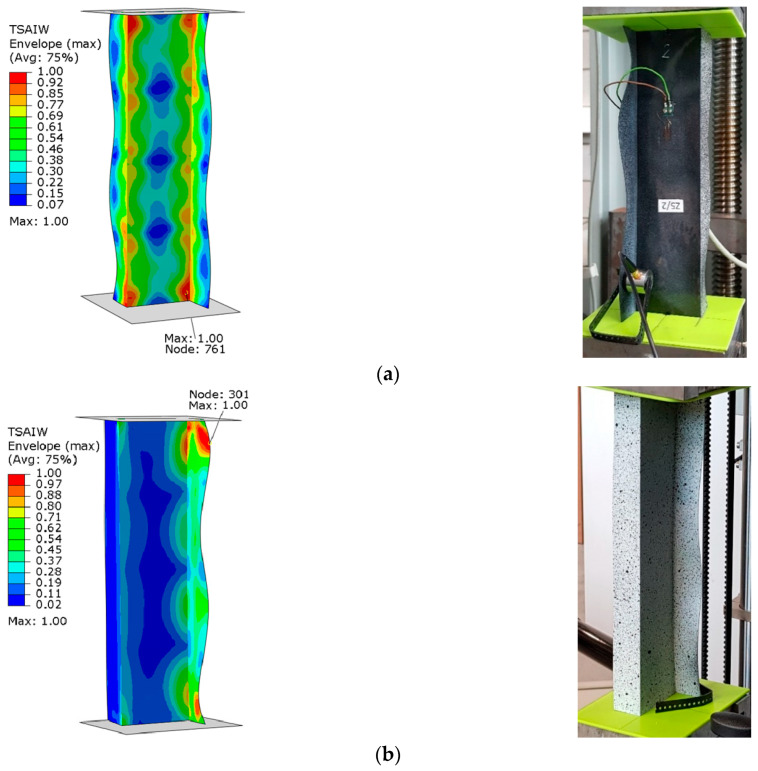
Column z_4—maps of the Tsai–Wu critical parameter and forms of post-critical deformation: (**a**) Test 1 and (**b**) Test 3.

The graphs presented show the value of the load initiating damage to the composite structure of the real structure, corresponding to the recorded increased value of the EA amplitude (triangles). The determined load values were confronted with the results of numerical analyses, which are marked on the diagrams with a blue dotted line drawn parallel to the time axis. For the numerical analysis, maps of the critical parameter corresponding to the failure criterion were determined. Figure 11, Figure 12, Figure 13 and Figure 14 present the areas where damage initiation of the first composite layer occurred, corresponding to the achievement of the value 1 of the critical parameter determined according to the Tsai–Wu criterion. The results of numerical calculations were compared with experimental forms of deformation in order to verify the applied FEM models. In all analyzed cases, the experimental and numerical forms of deformation were consistent.

The results obtained from the numerical analyses of the critical parameter map allowed us to identify the areas where there was a high probability of damage to the composite material structure. The analysis of the maps presented showed that these were mainly areas located close to the end sections of the analyzed Z-columns. Depending on the value of the load eccentricity, the area of initiation of the first layer appears on the web: Test 1 and on the profile wall of Test 2.

Table 4 presents the experimental and numerical values of the load initiating composite failure and the layer numbers (in brackets) where the critical value of failure parameter 1 was identified in the numerical computations. The results were compared for the analyzed Z-columns from z_1 to z_4.

The test results in Table 4 confirmed the high agreement between the value of the load initiating the failure of the composite material of the real structure and the corresponding value obtained for the numerical model. The maximum percent divergences were as follows: column z_1, 1.7%; column z_2, 8%; column z_3, 6%; and column z_4, 2.3%. This is a high level of agreement between the test results, confirming the correctness of the adopted test methodology.

On the basis of the test results obtained, it was noted that, irrespective of the configuration of the composite layers, the effect of the load eccentricity on the value of the force initiating the failure of the first layer is qualitatively the same for all considered columns with Z-shape cross-section. The realization of the load eccentricity in Test 3 resulted in a decrease in the composite-failure-initiating force. The percentage values of the load initiating composite failure in relation to the axial compression case are presented in Table 5. The quantitative analysis of the results obtained showed that the highest decrease in the value of the force initiating composite failure occurred in the case of laminate configuration z_4 and amounts to 85%, while the lowest decrease at the level of 76% was characterized by configuration z_3.

Producing structures from carbon–epoxy composites is expensive, making this type of research out of the reach of the researcher. In the author’s opinion, FEM modeling [76,77,78,79] may be of interest for considering progressive failure and failure processes in composites [80,81,82,83,84,85,86,87,88]. For this, future research directions will focus on the analysis of the covered state over the full range of loads leading to complete failure.

## 7. Conclusions

The study investigated the influence of compressive load eccentricity on the post-buckling behavior of thin-walled composite Z-columns. A combined qualitative and quantitative study of the influence of compressive load eccentricity on the post-buckling state up to load initiating the failure of the first composite layer was performed. The postcritical equilibrium paths of the structure (load deflection) determined in the tests showed a significant decrease in the stiffness of the structure due to the application of load eccentricity values of Test 2 and Test 3 compared to axial attrition of Test 1. The analysis of the Z-columns confirmed the high influence of the applied eccentricity of the compressive load on the working of the structure in the postcritical state. It should be noted that the highest stiffness of the Z-column was achieved during the axial force (ideal loading conditions, Test 1). The decrease in the stiffness of the structure z_1÷z_4 caused by the load eccentricity in the direction of axis 0° Test 2 and amounted to 12%÷22%. Significantly negative effects on the working of the structure were observed in the case of implementation of the load eccentricity in the 90° axis direction (Test 3). The existence of such a load eccentricity weakened the stiffness of the analyzed Z-columns z_1÷z_4 in the range of 46%÷64%. In addition, the results showed that the eccentricity of loading realized at the points: Test 2 and Test 3 had no change in the form of the postcritical deformation in relation to the compressive axial test (Test 1). The results from using the acoustic emission method (AEM) and the finite element method (FEM) showed agreement between the experimental and numerical results of the loads initiating composite failure. The applied test methodology allowed us to determine the effect of compression load eccentricity on the value of the load initiating composite structure failure. It was confirmed that the realization of load eccentricity for Test 3 causes a decrease in the value of the load initiating composite failure in comparison with axial compression (Test 1). This significantly leads to a reduction in the strength of the compressed structure. The observed reduction in the value of the load initiating composite failure was at the level of 76÷85%. The occurrence of such load eccentricity significantly weakened the strength of the compressed column. It could potentially result in premature failure of the CFRP material and ultimately reduce the load-carrying capacity of the construction. This is a dangerous effect, especially in the range of maintenance loads. The results obtained from this research have practical relevance, especially in the designing of thin-walled composite constructions, which are subject to unexpected inaccuracies that can significantly affect the performance.

## Figures and Tables

**Table 1:** Composite layup configurations.

Specimen	Configuration
z_1	[0/-45/45/90]s
z_2	[90/-45/45/0/]s
z_3	[45/-45/90/0/]s
z_4	[90/0/90/0]s

**Table 2:** Mechanical properties of CFRP composite.

*E_1_ (0°)*	*E_2_ (90°)*	*G_1,2_*	*ν_12_*	*F_T_* *_1_ (0°)*	*F_T2_ (90°)*	*Fs (45°)*	*F_C1_ (0°)*	*F_C2_ (90°)*
GPa	MPa	MPa	-	MPa	MPa	MPa	MPa	MPa
143	5826	3846	0.36	2221	49	84	641	114

**Table 3:** Changes in column stiffness—axis load to the eccentricity of the load.

Difference	Column—z_1		Column—z_2		Column—z_3		Column—z_4	
	EXP	FEM	EXP	FEM	EXP	FEM	EXP	FEM
Test 1 to Test 02	12%	8%	22%	19%	21%	18%	20%	19%
Test 1 to Test 3	46%	43%	64%	61%	58%	55%	53%	52%

**Table 4:** The failure-initiation force value for composite configuration.

		Test 1	Test 3
z_1	EXP	7374 N	1497 N
	FEM	7496 N (7)	1504 N (2)
	difference	1.60%	0.50%
z_2	EXP	7992 N	1349 N
	FEM	8229 N (2)	1466 N (1)
	difference	2.90%	8%
z_3	EXP	7417 N	1762 N
	FEM	7802 N (8)	1875 N (7)
	difference	4.90%	6%
z_4	EXP	9141 N	1349 N
	FEM	9301 N (2)	1381 N (7)
	difference	1.70%	2.30%

**Table 5:** Change in composite failure initiation load as a function of maximum compression load eccentricity.

*Difference*	*Sample—z_1*		*Sample—z_2*		*Sample—z_3*		*Sample—z_4*	
	EXP	FEM	EXP	FEM	EXP	FEM	EXP	FEM
Test 1 to Test 3	79.7%	79.9%	83.1%	82.2%	76.2%	76%	85.2%	85%

## Data Availability

Not applicable.
